# *De novo* Assembly and Characterization of the Global Transcriptome for *Rhyacionia leptotubula* Using Illumina Paired-End Sequencing

**DOI:** 10.1371/journal.pone.0081096

**Published:** 2013-11-21

**Authors:** Jia-Ying Zhu, Yong-He Li, Song Yang, Qin-Wen Li

**Affiliations:** Key Laboratory of Forest Disaster Warning and Control of Yunnan Province, Southwest Forestry University, Kunming, China; University of California, Riverside, United States of America

## Abstract

**Background:**

The pine tip moth, *Rhyacionia leptotubula* (Lepidoptera: Tortricidae) is one of the most destructive forestry pests in Yunnan Province, China. Despite its importance, less is known regarding all aspects of this pest. Understanding the genetic information of it is essential for exploring the specific traits at the molecular level. Thus, we here sequenced the transcriptome of *R. leptotubula* with high-throughput Illumina sequencing.

**Methodology/Principal Findings:**

In a single run, more than 60 million sequencing reads were generated. *De novo* assembling was performed to generate a collection of 46,910 unigenes with mean length of 642 bp. Based on Blastx search with an E-value cut-off of 10^−5^, 22,581 unigenes showed significant similarities to known proteins from National Center for Biotechnology Information (NCBI) non-redundant (Nr) protein database. Of these annotated unigenes, 10,360, 6,937 and 13,894 were assigned to Gene Ontology (GO), Clusters of Orthologous Group (COG), and Kyoto Encyclopedia of Genes and Genomes (KEGG) databases, respectively. A total of 5,926 unigenes were annotated with domain similarity derived functional information, of which 55 and 39 unigenes respectively encoding the insecticide resistance related enzymes, cytochrome P450 and carboxylesterase. Using the transcriptome data, 47 unigenes belonging to the typical “stress” genes of heat shock protein (Hsp) family were retrieved. Furthermore, 1,450 simple sequence repeats (SSRs) were detected; 3.09% of the unigenes contained SSRs. Large numbers of SSR primer pairs were designed and out of randomly verified primer pairs 80% were successfully yielded amplicons.

**Conclusions/Significance:**

A large of putative *R. leptotubula* transcript sequences has been obtained from the deep sequencing, which extensively increases the comprehensive and integrated genomic resources of this pest. This large-scale transcriptome dataset will be an important information platform for promoting our investigation of the molecular mechanisms from various aspects in this species.

## Introduction

The pine tip moth, *Rhyacionia leptotubula* (Lepidoptera: Tortricidae), is an important forestry pest posed a serious threat to *Pinus yunnanensis* and *Pinus armandii*, which distributed widely over much of Yunnan province in China [1]. It damages hosts in its larval stage. The larvae feed primarily on needles soon after hatching, and then bore into the shoots, causing severe deformation of host trees and significant long-term growth loss [2]. *R. leptotubula* infestations have been reported with 40% damage ratio in the serious outbreak area and a migrating speed of about 5 km to other regions per year [3]. In addition, the distributional areas of *R. leptotubula* are with large differentially ecological environments, indicating that this pest has the strong ability to overcome the diversifiable environmental stress. This will lead to the widespread outbreak of this pest in potential areas. In fact, *R. leptotubula* has been one of the most problematic plagues among forestry pests that threaten ecological safety of Yunnan. Despite its importance, there is still no effective strategy to control it. Currently, the control of this pest is mainly depended on the use of large quantities of pesticides, which not only costs a lot of money but also causes environmental pollution and insecticide resistance within the insect. The accumulation of considerable body of scientific knowledge about this pest is critical for designing suitable control tactics. However, *R. leptotubula* has been rarely studied from biological or ecological perspectives [4]. Also, very little is known about this pest at the molecular level. Such studies would provide fundamental data for deeply understanding the life histories and elucidating the molecular mechanism of *R. leptotubula* in forestry ecological systems.

In recent years, next generation sequencing has provided fascinating opportunities to effectively discover genes and explore genomic sequence resources in non-model organisms [5]. This technology has recently enabled the application of functional genomics to a broad range of insect species [6], [7]. In this study, we used the Illumina sequencing to build the transcriptome database for *R. leptotubula*. In a single run, we identified 60,572,936 raw reads assembled into 46,910 unigenes. The functional quality of the transcriptome was assessed by identifying the heat shock protein (Hsp) genes related to defense mechanisms of thermal stress. Additionally, a total of 1,450 simple sequence repeats (SSRs) also have been developed based on these unigenes. Results obtained from this study dramatically increase the genomic information for this species, and have potentially contributed to improve our understanding of this pest at the molecular level.

## Materials and Methods

### Ethics statement

Regarding to the field study, no specific permits were required. The location is not privately-owned or protected in any way. The field studies did not involve endangered or protected species.

### Insects

*R. leptotubula* larvae were collected from Zhehai forestry centre, Huize County, Yunnan Province, China. The samples were frozen at −80°C until use.

### cDNA library construction and sequencing

Total RNA was isolated using Trizol reagent (Invitrogen) following the manufacturer's instructions. The RNA integrity and quantity were determined on an Agilent 2100 Bioanalyzer (Agilent). Beads with oligo(dT) were used to isolate poly(A) mRNA. Then, mRNA was interrupted into short fragments by fragmentation buffer. Taking the fragments as templates, random hexamer-primer was used to synthesize the first-strand cDNA. After second strand cDNA synthesis, fragments were end repaired, a-tailed and indexed adapters were ligated. After products fractionated by agarose gel electrophoresis, suitable fragments were selected and enriched with PCR to create the final cDNA library. At last, library was sequenced using Illumina HiSeq™ 2000.

### Assembly of Illumina reads

Before the transcriptome assembly, a stringent filtering process of raw sequencing reads was carried out to discard the dirty reads. The dirty reads include reads with adaptors, unknown nucleotides larger than 5% and low quality (the number of bases with quality value ≤10 more than 20%). The reads obtained were randomly clipped into 21 bp K-mers for assembly, which was assessed to provide the best result for transcriptome assembly *De novo* assembly was carried out with short reads assembling program-Trinity [8]. During assembly, the minimum contig length and pair number cutoff of Trinity were respectively set as 100 bp and 4, and other default parameters were used to run it. Firstly, clean reads with certain length of overlap were combined to form longer fragments, contigs. Then, the reads were mapped back to contigs; with paired-end reads it is able to detect contigs from the same transcript as well as the distances between these contigs. Finally, contigs were connected to get sequences that cannot be extended on either end. These sequences were termed as unigenes. Unigenes were submitted to Blastx search (E-value<10^−5^) against protein databases including National Center for Biotechnology Information (NCBI) non-redundant protein (Nr), Swiss-Prot, Kyoto Encyclopedia of Genes and Genomes (KEGG) and Cluster of Orthologous Groups (COG). The best aligning results were used to decide sequence direction of unigenes. If results of different databases conflict with each other, a priority order of alignments from the Nr, Swiss-Prot, KEGG and COG databases was followed to decide the sequence direction. When a unigene happens to be unaligned to none of the above databases, ESTScan software [9] was used to decide its direction.

### Functional unigene annotation

Unigenes were firstly aligned by Blastx (E-value<10^−5^) to protein databases of Nr, Swiss-Prot, KEGG and COG, retrieving proteins with the highest sequence similarity with the given unigenes along with their protein functional annotations. In the Blastx search step, the best aligning results were defined according to the feedback E-value. And the first hits were used to the following analysis. Functional annotation by Gene Ontology (GO) terms was analyzed by Blast2Go software [10]. Unigenes were also aligned to the COG database to predict and classify possible functions. Pathway annotation was performed using Blastall software against the KEGG database. Homologous protein domains from translated *R. leptotubula* transcriptomic sequences were identified by searching against the Pfam database using HMMER3 [11]. *R. leptotubula* Hsp genes were searched with Lepidopteran Hsps as queries using BlastX in the free software BioEdit program. Multiple amino acid sequence alignments were conducted with ClustalX (v1.83) program [12]. Phylogenetic tree was constructed from the multiple alignments using MEGA 5.2.1 software [13] using a maximum parsimony method with 1000 bootstrap replications.

### Development and detection of SSR markers

Potential SSR markers were detected among unigenes using the MISA tool (http://pgrc.ipk-gatersleben.de/misa). The parameters were adjusted for identification of perfect mono-, di-, tri-, tetra-, penta-, and hexanucleotide motifs with a minimum of 12, 6, 5, 5, and 4 repeats, respectively. Based on MISA results, Primer3 v2.23 (http://primer3.sourceforge.net) was used to design the primer pairs following the criteria described in Wang et al. [14]. In total, 30 pairs of primers were randomly selected and validated by PCR reactions. The PCR program was as follows: initial denaturation for 5 min at 94°C followed by 35 cycles of 30 s at 94°C, 30 s at 55–60°C, and 30 s at 72°C, with a final extension at 72°C for 10 min. The PCR products were analyzed by electrophoresis.

### Data deposition

The cleaned short read sequences were deposited in the DNA Data Bank of Japan (DDBJ) (http://www.ddbj.nig.ac.jp/) Sequence Read Archive under the accession number DRA001033. The *de novo* assembly sequence data is available from Jia-Ying Zhu on request (jyzhu001@gmail.com).

## Results and discussion

### Sequencing and *de novo* assembly

In a single run, a total of 60,572,936 raw reads with the length of 90 bp were generated. After filtering, 54,476,454 clean reads in total length of 4,902,880,860 bp with 97.27% Q20 bases (those with a base quality greater than 20) were obtained. The GC content of them was 50.53%, which is comparable to that value of other insect and eukaryote sequencing projects [15]. Using the Trinity assembling program, clean reads were assembled into 111,786 contigs and 46,910 unigenes, with average lengths of 292 and 642 bp (Table 1). The obtained a large number of unigenes in this study is consistent with other studies based on Illumina technology [16], of which average contig and unigene lengths of the assembly are less than that of the 454-pyrosequencing assembly [17]. The unigene length distribution followed the contig distribution closely, with the majority being shorter sequences. The length distribution of them indicated that above 85% contigs or unigenes were between 100 and 1,000 bp in length. The N50 values of these contigs and unigens were 424 and 888 bp, respectively. Although the majority of the contigs and unigenes were in relatively short length, we obtained 5,151 contigs and 6,664 unigenes which were greater than 1000 bp in length. The assembly results indicated that the length distribution pattern and mean length of contigs and unigenes was similar to those in the previous Illumina-transcriptome studies [18], [19], suggesting that the transcriptome sequencing data from *R. leptotubula* was effectively assembled.

**Table 1:** Summary of assembled contigs and unigenes.

	Contigs	Unigenes
100–500 bp	97,874	30,292
501–1,000 bp	8,761	9,953
1,001–1,500 bp	2,793	3,357
1,501–2,000 bp	1,272	1,629
2,001–2,500 bp	523	720
2,501–3,000 bp	262	371
>3,000 bp	301	587
Total number	111,786	46,910
Total length (bp)	32,593,120	30,109,909
Mean length (bp)	292	642
N50 (bp)	424	888

### Unigene annotation

**Figure 1:**
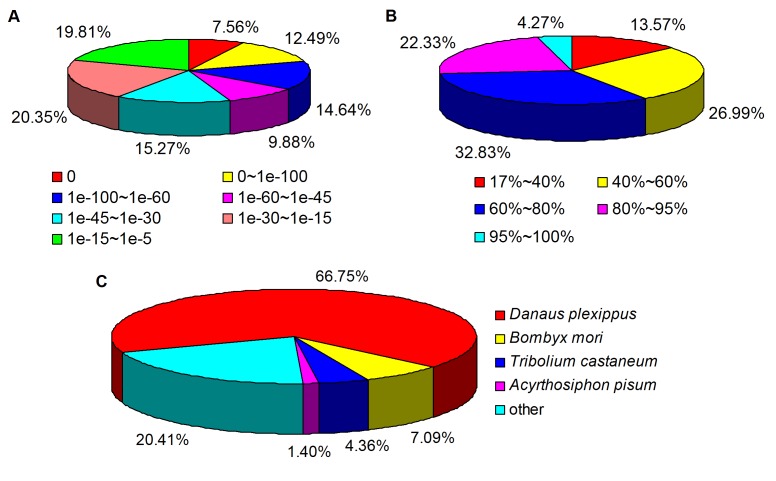
Characteristics of similarity search of unigenes against Nr databases. (A) E-value distribution of BLAST hits for each unigene with a cutoff E-value of 1E-5 in the Nr database. (B) Similarity distribution. (C) Species distribution. The distribution is shown as a percentage of the total BLAST hits for each unigene with a cutoff E-value of 1E-5 in the Nr database.

Unigenes were firstly interrogated against the NCBI Nr protein database using a Blastx E-value threshold of 10^−5^. Of 46,910 unigenes, 22,580 (48.13%) had Blast hits to known proteins (Table S1). A significant percentage of transcripts (near half of all unigenes) were found to be unique to *R. leptotubula*, which perhaps could be attributed to the presence of novel genes. In addition, due to the lack of a reference genome, it is kind of difficult to estimate the number of genes and predict the potential functions of the transcripts [20]. This result is consistent with previous observations that extremely low levels of conservation occurs between insect genomes [21], suggesting that *de novo* sequencing and assembling efforts will be necessary for most insect species, even when sequence data are available for other members of the same order [22]. The length of query sequences was crucial in determining the level of significance of the Blast match. The proportion of unigenes with significant Blast scores increased sharply from 500–1000 bp to 1000–1500 bp. The result indicates that the proportion of sequences with matches in the Nr database is greater among the longer assembled sequences, which is similar to other analytical results of the next-generation transcriptome [23]. The E-value distribution of the top hits in the Nr database showed that 20.05% of the sequences have strong homology (smaller than 1E-60), whereas 60.14% of the homolog sequences ranged from 1E-15 to 1E-60 (Figure 1A). On the other hand, the similarity distribution demonstrated that 26.6% of the unique sequences with best hits have a similarity higher than 80%, while 59.82% of the hits have a similarity ranging from 40% to 80% (Figure 1B). Homologous genes come from several species, with 66.75% of the unigenes had the highest homology to genes from *Danaus plexippus*, followed by *Bombyx mori* (7.09%), *Tribolium castaneum* (4.36%), and *Acyrthosiphon pisum* (1.40%) (Figure 1C). The highest percentage of the unigenes matched to the genes of *D. plexippus* may be due to that *R. leptotubula* is phylogenetically closer to *D. plexippus* than other species, and the genome information of *D. plexippus* is now available [24], providing sufficient gene sequences and annotations for comparison analyses.

GO is an annotation framework that provides a standardized vocabulary that is used to assign function to uncharacterized sequences [25]. Using Blast2GO, 33,862 terms for biological process category, 20,319 for cellular component category, and 11,582 for molecular function category were produced (Table 2). In total, 10,360 unigenes (22.8% of all unigenes) were assigned to GO term annotations (Table S2). The low proportion of *R. leptotubula* unigenes with GO annotation is possibly due to large number of uninformative gene descriptions of the protein database hits. These 65,763 GO terms were summarized into 54 sub-categories. Among the biological process category, cellular process (18.08%), metabolic process (14.02%), biological regulation (7.69%), multicellular organismal process (7.17%) and regulation of biological process (7.04%) were the most dominant subcategories. In cellular component category, the four most common categories were cell (28.84%), cell junction (28.14%), organelle (17.09%), and organelle part (10.17%). Binding (39.22%) and catalytic activity (41.20%) were the most highly represented subcategories in molecular function category. Similar observations for metabolic processes were reported in other insect's tanscriptomes [6], [26].

**Table 2:** GO classifications of assembled unigenes.

Ontology	Class	Number of unigenes	Percent of unigenes
Biological process	biological adhesion	250	0.74
	biological regulation	2605	7.69
	carbohydrate utilization	1	0.00
	cell killing	4	0.01
	cell proliferation	179	0.53
	cellular component organization or biogenesis	1983	5.86
	cellular process	6123	18.08
	death	259	0.76
	developmental process	2359	6.97
	establishment of localization	1547	4.57
	growth	300	0.89
	immune system process	220	0.65
	localization	1800	5.32
	locomotion	438	1.29
	metabolic process	4749	14.02
	multi-organism process	215	0.63
	multicellular organismal process	2428	7.17
	negative regulation of biological process	714	2.11
	pigmentation	57	0.17
	positive regulation of biological process	575	1.70
	regulation of biological process	2385	7.04
	reproduction	691	2.04
	reproductive process	681	2.01
	response to stimulus	1890	5.58
	rhythmic process	69	0.20
	signaling	1314	3.88
	viral reproduction	26	0.08
Cellular component	cell	5859	28.84
	cell junction	174	0.86
	cell part	5717	28.14
	extracellular region	130	0.64
	extracellular region part	127	0.63
	macromolecular complex	1885	9.28
	membrane-enclosed lumen	667	3.28
	organelle	3473	17.09
	organelle part	2066	10.17
	synapse	118	0.58
	synapse part	97	0.48
	virion	3	0.01
	virion part	3	0.01
Molecular function	antioxidant activity	36	0.31
	binding	4543	39.22
	catalytic activity	4772	41.20
	channel regulator activity	9	0.08
	electron carrier activity	2	0.02
	enzyme regulator activity	274	2.37
	metallochaperone activity	3	0.03
	molecular transducer activity	280	2.42
	nucleic acid binding transcription factor activity	250	2.16
	protein binding transcription factor activity	113	0.98
	receptor activity	197	1.70
	structural molecule activity	454	3.92
	translation regulator activity	7	0.06
	transporter activity	642	5.54

A total of 6,937 unigenes were annotated by three categories: biological process,cellular component, and molecular function. The number and ratio of unigenes assigned to level 2 GO terms were shown. Percent of unigenes represents each class comprising in all unigenes assigned into each ontology category.

**Figure 2:**
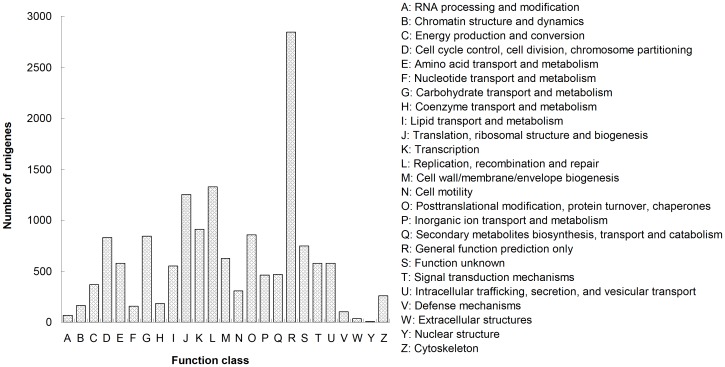
Histogram presentation of COG classification. In total, 15,082 unigenes were grouped into 24 COG classifications.

To further annotate their functions, unigenes were aligned to COG database to find homologous genes. In total, 6,937 unigenes (14.80%) were annotated into 15,082 COG terms, which were formed 25 classifications (Figure 2). As each categorized COG term represents an ancient conserved domain, the result indicated that only a small proportion of the putative proteins that encoded by the assembled unigenes carried protein domains with annotation for COG categories [27]. Among the functional classes, the cluster for general function prediction only (18.87%) was the largest group, followed by replication, recombination and repair (8.80%), transcription (8.29%), carbohydrate transport and metabolism (5.58%), and cell cycle control, cell division, chromosome partitioning (5.48%). Only few sequences were present in nuclear structure (0.04%), extracellular structures (0.21%), and RNA processing and modification (0.43%), represented the smallest groups.

To evaluate the molecular interaction and reaction networks of the unigenes, they were compared with KEGG and the corresponding pathways were established. A total of 13,894 unigenes (29.62%) were annotated in KEGG and located to 242 known KEGG pathways (Table S3). Except for lipoic acid metabolism, butirosin and neomycin biosynthesis, D-glutamine and D-glutamate metabolism, thiamine metabolism, biotin metabolism, allograft rejection, graft-versus-host disease, polyketide sugar unit biosynthesis, and asthma, all other pathways were associated with more than 5 unigenes. A total of 2,479 unigenes (17.84%) were assigned to the metabolic pathways, composing the largest group, indicating that the active metabolic processes were underway in the larval stage of *R. leptotubula*. In addition to the unigenes assigned to the metabolism pathways, the well represented pathways were followed by purine metabolism (3.79%), RNA transport (3.66%), and spliceosome (3.33%). The results of GO, COG and KEGG annotations provide a valuable resource for investigating specific processes, functions and pathways that will guide research on *R. leptotubula*.

### Protein domains

**Figure 3:**
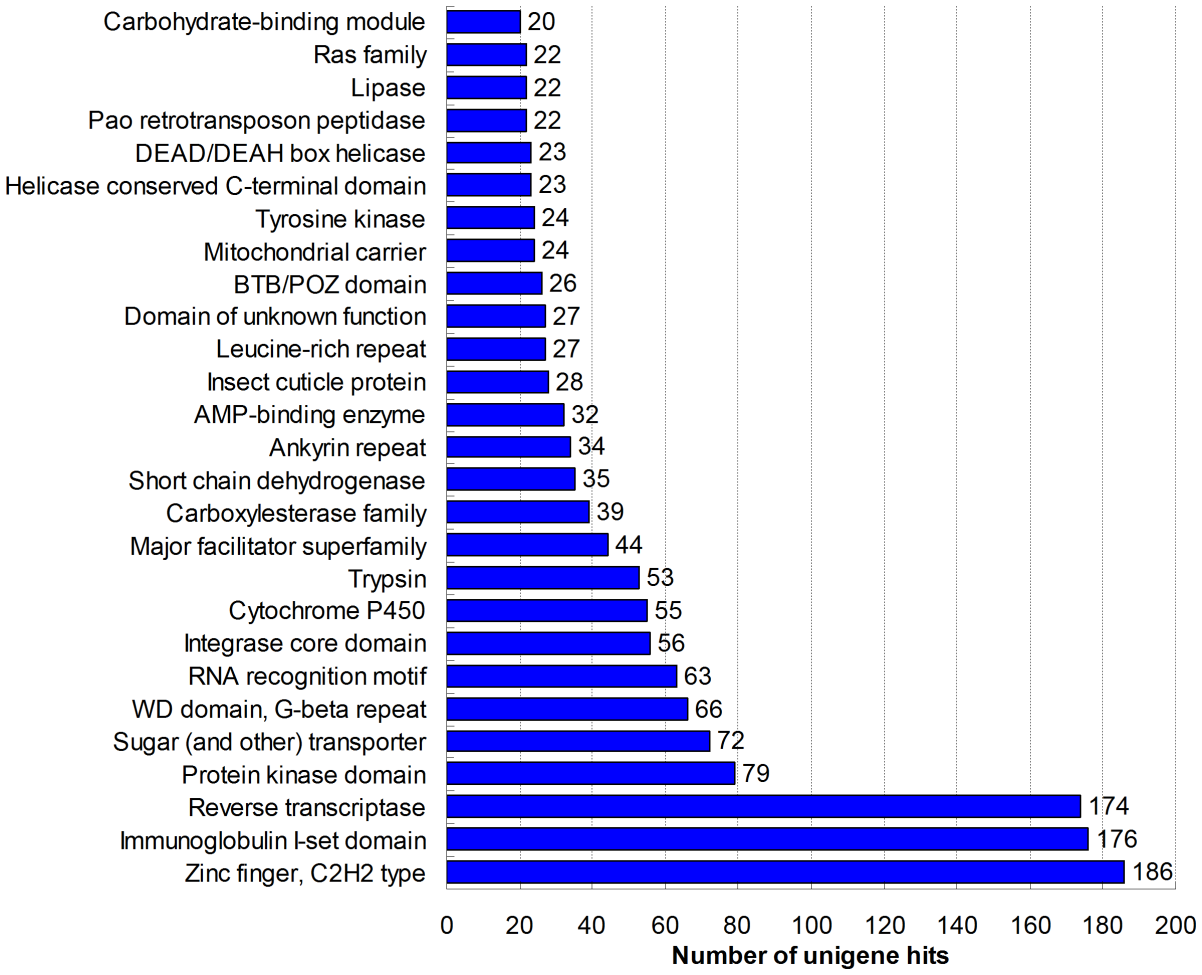
Top 27 Pfam domains predcted in translated unigenes.

Pfam domains were identified in 5,926 unigenes (12.63% of all unigenes) (Table S4). A total of 1,916 protein domains were identified. Most domains were found containing 1–3 sequences, with a small proportion appearing more frequently. It is similar to that of other transcriptome data [28]. A summary of the most frequent classifications containing ≥20 number of unigene hits was shown in Figure 3. The most prevalent domains were reverse transcriptase, immunoglobulin I-set domain and zinc finger, C2H2 type. Moreover, highly represented domains were RNA recognition motif, WD repeat, transcription and proliferation, sugar (and other) transporter, and protein kinase domain. Cytochrome P450s and carboxylesterases involved in the metabolism of endogenous compounds including juvenile hormones, ecdysteroids and pheromones, and xenobiotics such as drugs, pesticides, plant toxins, chemical carcinogens and mutagens, were also dominant [29], [30]. There were 55 and 39 unigenes corresponding to cytochrome P450 and carboxylesterase, respectively. P450 enzymes metabolize insecticides, resulting in the enhanced detoxification in many insects, hence, the development of insecticide resistance [31], [32]. Also, carboxylesterases have been known to be associated with insecticide resistance and detoxification, which often mediate resistance to organophosphates, carbamates, and to a lesser extent, pyrethroids [33]. The future characterization of these gene families encoding gene products involved in insecticide resistance may provide a potential molecular basis for resistance in this species.

### Identification of Hsp genes

**Figure 4:**
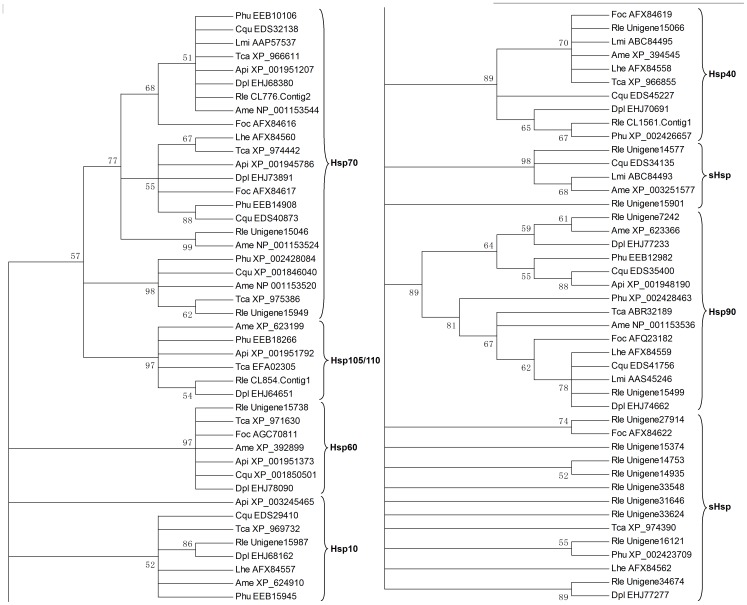
Phylogenetic analysis of heat shoch protens from different insects. Analyses were performed using only sequences predicted to encode complete ORFs. The tree is a 50% consensus tree. Abbreviations are as follows: Api, *Acyrthosiphon pisum*; *Ame, Apis mellifera*; Cqu, *Culex quinquefasciatus*; Dpl, *Danaus plexippus*; Foc, *Frankliniella occidentalis*; Lmi, *Locusta migratoria*; Lhe, *Lygus hesperus*; Phu, *Pediculus humanus corporis*; Rle, *Rhyacionia leptotubula*; Tca, *Tribolium castaneum*.

Hsps are a superfamily that has been widely studied in a wide range of organisms. In addition to act as molecular chaperons, promoting correct refolding and preventing aggregation of denatured proteins in response to various stress factors, Hsps also play important role in diverse physiological and biological processes, including embryogenesis, diapause, and morphogenesis [34], [35]. Based on their molecular weights, they are usually assigned to several families, including Hsp10, small Hsp (sHsp), Hsp40, Hsp60, Hsp70, Hsp90 and Hsp105/110 [36], [37]. It is well known that both prokaryotic and eukaryotic cells respond to unfavourable environmental conditions by most predominantly increased synthesis of Hsps [38]. In order to elucidate the molecular basis of environmental stress tolerance in *R. leptotubula*, unigenes that encode Hsps were sought in the transcriptome. A total of 47 Hsp related unigenes were identified, which were segregated into clades corresponding to 6 Hsp families: Hsp10, sHsp, Hsp40, Hsp60, Hsp70, Hsp90 and Hsp105/110 (Table 3 and Figure 4). Of these, 21 were found to represent full length open reading frames (ORFs).

**Table 3:** Putatively identified heat shock protein (Hsp) genes.

Hsp family	Unigen ID	Length (bp)	First hit	Identity (%)	E_value	Organism	Blast annotation
Hsp10	Unigene15987	610	EHJ68162	84	1.00E-44	*Danaus plexippus*	putative 10 kDa heat shock protein
sHsp	Unigene4122	229	AAV48822	66	8.00E-22	*Venturia canescens*	small heat shock protein
	Unigene8202	319	EHJ69640	47	7.00E-12	*Danaus plexippus*	heat shock protein hsp23.7
	Unigene14577	1400	ADK55519	99	6.00E-100	*Spodoptera litura*	small heat shock protein
	Unigene14753	1239	AAZ14793	69	1.00E-107	*Choristoneura fumiferana*	33.6 kDa small heat shock protein
	Unigene14935	779	ADK55523	67	2.00E-83	*Spodoptera litura*	small heat shock protein
	Unigene15374	672	ADO33017	69	2.00E-67	*Biston betularia*	heat shock protein 19.4
	Unigene15901	928	EHJ73481	42	9.00E-44	*Danaus plexippus*	heat shock protein 25.4
	Unigene16121	1004	AAV91360	86	5.00E-94	*Lonomia obliqua*	heat shock protein 1
	Unigene27914	613	EHJ77276	57	1.00E-52	*Danaus plexippus*	small heat shock protein
	Unigene28691	257	EHJ70499	58	1.00E-19	*Danaus plexippus*	heat shock protein hsp23.7
	Unigene31646	718	ADX96000	94	3.00E-91	*Cydia pomonella*	small heat shock protein 19.8
	Unigene31998	446	NP_001164470	88	6.00E-64	*Bombyx mori*	19.5 kDa heat shock protein
	Unigene33548	687	BAE94664	81	8.00E-80	*Chilo suppressalis*	small heat shock protein 19.7
	Unigene33624	705	NP_001037038	78	8.00E-81	*Bombyx mori*	heat shock protein 20.4
	Unigene34674	727	ADX96001	88	1.00E-87	*Cydia pomonella*	small heat shock protein 19.9
	Unigene5650	792	EHJ69640	40	4.00E-31	*Danaus plexippus*	heat shock protein hsp23.7
	CL2110.Contig2	573	EHJ73481	34	9.00E-16	*Danaus plexippus*	heat shock protein 25.4
Hsp40	Unigene15066	1649	EHJ65096	92	0	*Danaus plexippus*	DnaJ-5 (HSP40)
	CL1561.Contig1	1552	NP_001040292	87	0	*Bombyx mori*	DnaJ (Hsp40) homolog 2
Hsp60	Unigene5044	340	EHJ77502	71	9.00E-39	*Danaus plexippus*	63 kDa chaperonin, mitochondrial
	Unigene15738	2124	ACT52824	95	0	*Chilo suppressalis*	heat shock protein 60
	Unigene19939	537	EHJ78090	72	2.00E-61	*Danaus plexippus*	heat shock protein 60
	Unigene30172	422	ADM13383	76	3.00E-54	*Polypedilum vanderplanki*	heat shock protein 60
Hsp70	Unigene2264	206	ACQ78180	71	1.00E-19	*Spodoptera exigua*	heat shock protein 70
	Unigene10792	307	EHJ78227	76	5.00E-11	*Danaus plexippus*	heat shock 70 kDa protein 14
	Unigene13413	240	ACD84945	100	5.00E-37	*Macrocentrus cingulum*	heat shock cognate protein 70
	Unigene15046	3369	AAN86047	97	0	*Spodoptera frugiperda*	heat shock cognate 70 protein
	Unigene15949	4237	ACM78945	94	0	*Spodoptera exigua*	heat shock protein 70
	Unigene16976	222	AAN73310	100	2.00E-35	*Cotesia rubecula*	heat-shock protein 70
	Unigene18794	1236	EHJ78227	80	0	*Danaus plexippus*	heat shock 70 kDa protein 14
	Unigene19309	428	EFN65945	88	1.00E-60	*Camponotus floridanus*	heat shock 70 kDa protein cognate 4
	Unigene21400	354	AEI58996	98	2.00E-41	*Bombyx mori*	heat shock protein 70B
	Unigene26670	258	ABD67500	91	5.00E-37	*Sphaeroforma arctica*	heat shock protein 70
	Unigene29332	266	ABV55505	94	1.00E-40	*Microplitis mediator*	heat shock protein 70
	Unigene29488	229	AAN73310	95	2.00E-34	*Cotesia rubecula*	heat-shock protein 70
	CL776.Contig1	277	EHJ68380	98	3.00E-44	*Danaus plexippus*	Heat shock 70 kDa protein cognate 4
	CL776.Contig2	2113	Q9U639	98	0	*Manduca sexta*	heat shock cognate 70 protein
	CL2709.Contig1	304	AAO65964	93	5.00E-47	*Manduca sexta*	heat shock protein 70
Hsp90	Unigene7242	2231	EHJ77233	81	0	*Danaus plexippus*	heat shock protein
	Unigene15499	2448	AFA35118	98	0	*Cydia pomonella*	heat shock protein 90, partial
	Unigene16067	247	ADK55517	92	7.00E-13	*Spodoptera litura*	heat shock protein 90 cognate
	Unigene16739	252	BAF75925	78	4.00E-31	*Physarum polycephalum*	heat shock protein 90
	Unigene26468	235	AAY67878	72	4.00E-25	*Pseudourostyla cristata*	heat shock protein 90
Hsp105/110	CL854.Contig1	2295	AEB26316	81	0	*Helicoverpa armigera*	heat shock protein 105
	CL854.Contig2	1800	AEB26316	85	0	*Helicoverpa armigera*	heat shock protein 105

Hsp10, a near 10 kDa chaperone, is analogous to the bacterial GroES subunit. It has been proven to be an essential component of the protein folding apparatus, which co-chaperones with Hsp60 for protein folding as well as the assembly and disassembly of protein complexes [39]. In the mitochondria, Hsp10 forms a heptameric lid, which binds to a double-ring toroidal structure comprising seven Hsp60 subunits per ring [40]. It originally identified as a predominantly mitochondrial chaperone, but has been found to localize to a number of cellular compartments [41]. Functionally besides participating in Hsp10/60 protein folding machine, Hsp10 is related to diverse physiological functions in mammals [42]. However, Hsp10 in insects has not been functionally defined in detail. Unigene 15987 encoding a complete Hsp10 ORF was identified in *R. leptotubula* transcriptome. The predicted Hsp10 amino acid sequence showed a moderate degree of identity (50% to 84%) to other insect Hsps (Figure S1). This is in agreement with previous work, which reported that Hsp10 is highly conserved [19].

The family of sHsp represents the proteins with low molecular weights of 12–43 kDa depending on the variable N- and C-terminal extensions, which contains a conserved alpha-crystallin domain (approximately 100 amino acid residues, a hallmark of the sHsp family). [43]. Functionally, sHsps associate with nuclei, cytoskeleton and membranes, and as molecular chaperones they bind partially denatured proteins, thereby preventing irreversible protein aggregation during stress [44]. They are ubiquitous in almost organisms studied and have been intensively studied in bacteria and plants. Different organisms have different numbers of sHsps, ranging from only one in yeast up to 30 in higher plants [45]. In *Bombyx mori*, 16 sHsps have been identified from the genome [46]. Here, 17 sHsps were predicted in the derived *R. leptotubula* sequences, of which 11 appeared to be with complete ORFs. Conserved domain search revealed that alpha-crystallin domain was positioned between N- and C-terminal extensions of *R. leptotubula* sHsps. sHsps are the most diverse in structure amongst the various families of stress proteins [47]. Amino acid sequence comparisons of *R. leptotubula* sHsps indicated that they were highly variable with identity varied from 7 to 20%. Also, they showed very poor sequence conservation to other insect's sHsps (Figure S2). As could be expected, *R. leptotubula* sHsps showed more similarity to other species in the C-terminal region, compared to the N-terminal region. Interestingly, phylogenetic relationship revealed that Unigene27914 and Unigene34674 were respectively conserved with *Frankliniella occidentalis* (AFX84622) and *Danaus plexippus* (EHJ77277) sHsps (Figure 4).

Hsp40 is a homologue of bacterial DnaJ protein. Thus, it is also named DnaJ. Hsp40s have been preserved throughout evolution and are important for protein translation, folding, unfolding, translocation, and degradation, primarily by stimulating the ATPase activity of Hsp70s [48], [49]. They have three distinct domains: J domain known to mediate interaction with Hsp70 and regulate its ATPase activity; glycine and phenylalanine-rich region (G/F domain) possibly acting as a flexible linker, and cysteine-rich region (C domain) containing 4 [CXXCXGXG] motifs resembling a zinc-finger like structure [50]. Depending on the presence of these domains, Hsp40 can be divided into 3 groups (type I, II and III) [51]. All types contain the J domain, but type III only has J domain. In addition to J domain, type I has G/F and C domain, and type II has G/F domain. In the *R. leptotubula* assembly, 2 unigenes (Unigene15066 and CL1561.Contig1) with complete ORFs produced the best sequence matches to Hsp40. Their deduced amino acid sequences were with 23–74% identity to the previously reported insect Hsp40s (Figure S3). Based on the domain structure, they were Type II Hsp40s.

Hsp60s are thought to be a co-chaperone for Hsp10, as essential chaperones required for the folding and multimeric complex assembly of mitochondrial proteins [52]. An important activity of Hsp60s is mediation of the native folding of proteins in an ATP-dependent manner, which typically represents by the highly conserved ATP binding motif in the sequence [53]. They are a group of proteins with distinct ring-shaped, or toroid (double doughnut) quaternary structures [54]. Classical Hsp40s localize mainly to the cytosol in bacteria, the chloroplasts in plants and the mitochondria in animals [55]. Those located in mitochondria are associated with mitochondrial targeting motifs. Four unigenes were found to encode Hsp40s. Of them, Unigene15738 was identified with full ORF. It contained classical mitochondrial Hsp60 signature motif (AAVEEGIVPGGG), indicating that it is mostly located in mitochondria. Hsp60s are evolutionary conserved across taxa. The deduced amino acid sequence of Unigene15738 was highly similar to that of other insects, exhibiting 73–88% identity (Figure S4).

Hsp70s function for facilitating the assembly of multimeric protein complexes and as molecular chaperons for facilitating intracellular folding of proteins, for secretion and transport, which generally interact with Hsp40s [56]. They seem to be the dominant protein expressed following most environmental insults [57]. In total, we obtained 12 Hsp70 related sequences. Among them, Unigene15046, Unigene15949 and CL776.Contig2 were with ORFs. Hsp70 family is broadly and highly conserved across prokaryotes and eukaryotes. The Hsp70 amino acid sequences of *R. leptotubula* were shown to be highly homologous (39–96% identity) to that of other insects (Figure S5). According to subcellular locations, HSP70 is divided into 3 subgroups (cytoplasm with C-terminal EEVD/E sequence, mitochondrion with MitoProt sequence and endoplasmic reticulum with KDEL sequence) in insects [58]. Based on the core signatures associated with each subgroup, Unigene15046, Unigene15949 and CL776.Contig2 encoded endoplasmic reticulum, mitochondrion and cytoplasm localized Hsp70, respectively.

Hsp90 is distinguished from other chaperones because of its association with specific proteins including various kinases, components of the cytoskeleton, elements of the protein synthesis machinery and intracellular receptors [59]. Hsp90 is a highly conserved molecular chaperone contributing to the folding, maintenance of structural integrity and proper regulation of a subset of cytosolic protein [60]. Five Hsp90-encoding genes were identified in the transcriptome of *R. leptotubula.* Unigene7242 and Unigene15499 contained complete ORFs. The core signatures or motifs were characterized in their deduced amino acid sequences [61]. A low degree of conservation (25% identity) was observed between the protein sequences encoding by these two unigenes. Ranged from 24% to 92% degree of conservation was observed between these two sequences as was the case for other insects Hsp90 (Figure S6). According to the subcellular location of Hsp90, the Hsp90 family is similar to Hsp70 that cytoplasmic, endoplasmic reticulum, and mitochondria types were present in insects [60]. Unigene15499 was cytoplasmic, while Unigene7242 was mitochondrial.

Hsp105/110 family is a divergent subgroup of the Hsp70 family. In mammals, the proteins of this family exist as complexes associated with Hsp70 (a constitutive form of Hsp70) and function to suppress the aggregation of denatured proteins in cells under severe stress, in which the cellular ATP level decreases markedly [62], [63]. Except for constitutive expression, Hsp105/110 is also induced by various stresses [64]. In insects, the role of Hsp105/110 has not been clearly defined. Blast analysis of the *R. leptotubula* transcriptome identified two sequences (CL854.Contig1 and CL854.Contig2) corresponding to gene products homologous with Hsp105/110. CL854.Contig1 was found to encode complete ORF. The translated amino acid sequence of CL854.Contig1 represented high conserved proteins, with sequence identities ranging from 51–77% to that of other insects (Figure S7).

### SSR marker identification and validation

**Figure 5:**
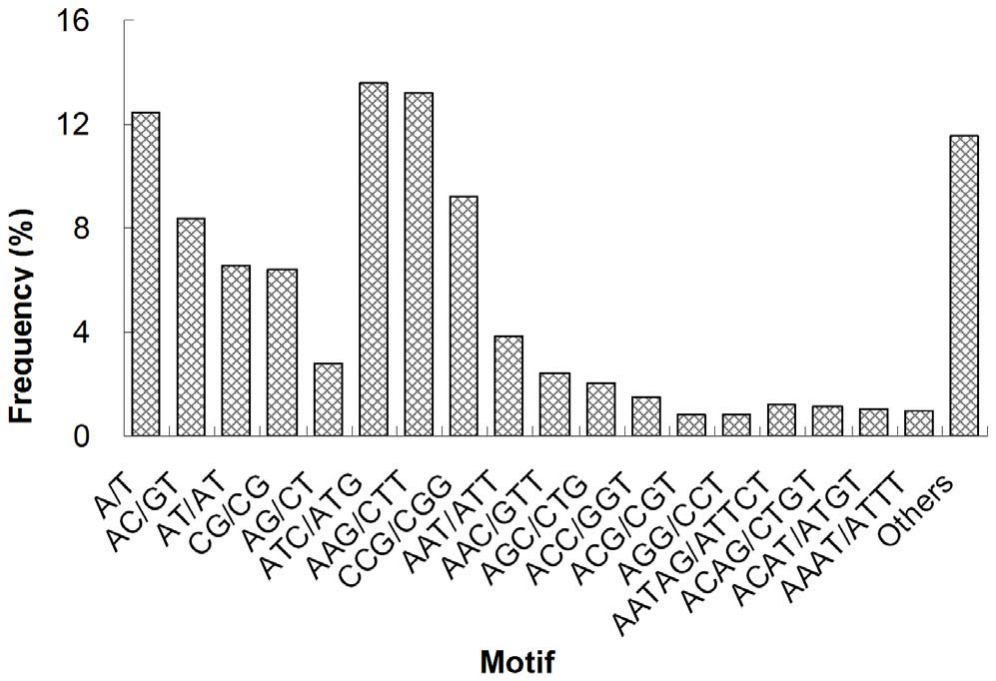
Frequency distribution of SSRs based on motif types. The frequency of main motif types was displayed.

Among various molecular markers, SSRs are highly polymorphic, very useful in various aspects of molecular genetic studies, including researches of genetic diversity assessment, comparative genomics, gene flow characterization, and genetic linkage mapping [65]. For development of molecular markers for *R. leptotubula*, all unigenes were used to mine potential microsatellites using MISA tool. In total, 1,370 sequences containing 1,450 SSRs were identified, with 78 of the sequences containing more than one SSR (Table 4). The SSR frequency in *R. leptotubula* transcriptome was 3.09%, and the distribution density was 20.77 per kb. The SSR frequency in this study is relatively lower than that of other insects belonging to other orders [66]. It is well known that isolation and characterization of microsatellite markers is clearly more difficult in Lepidoptera than in most other organisms, and very few microsatellite loci have been reported for Lepidoptera [67]. Given that the mono-nucleotide repeats may not be accurate because of the sequencing errors and assembly mistakes, 1,260 SSRs that exclude mono-nucleotide repeats were detected with the frequency of 2.69%, indicating the highly efficient discovery. As expected, most repeats (97.17%) were perfect repeats among the identified SSRs. As shown in Table 5, the repeat unit of potential SSRs mostly represented was 5 and 6, which accounting for 29.38% and 34.00%, respectively. In general, tri-nucleotide repeats have been observed to have the highest frequency [68]. In agreement with this, the tri-nucleotide repeats were the most abundant motif type (48.07%) (Figure 5). Of the tri-nucleotide repeat, ATC/ATG (13.59%), AAG/CTT (13.17%), and CCG/CGG (9.24%) were the dominant repeat motifs. The most abundant mono- nucleotide repeat type was A/T (12.48%). With respect to di-nucleotide repeat motif, AC/GT, AT/AT, CG/CG, and AG/CT were indentified in the database with frequencies of 8.34%, 6.55%, 6.41%, and 2.83%, respectively. In addition to those displayed in Figure 3, the frequency of the remaining 74 types of motifs accounted for 11.52%. Based on the indentified SSRs, 1,136 SSR primer pairs were designed (Table S5). A subset of 30 SSR primer pairs was randomly selected for validation of marker assay performance. Twenty-five primer pairs resulted in successful PCR amplification (Table S6). The results demonstrated that the potential detected SSRs in the dataset would be a wealth of resource for developing highly polymorphism SSR markers in *R. leptotubula*.

**Table 4:** Statistics of SSR searching results.

Item	Number
Total number of sequences examined	46910
Total size of examined sequences (bp)	30,109,909
Total number of identified SSRs	1450
Number of SSR containing sequences	1370
Number of sequences containing more than 1 SSR	78
Number of SSRs present in compound formation	41

**Table 5:** Distribution of SSRs based on the number of repeat units.

Number of repeats	Mono-	Di-	Tri-	Quad-	Penta-	Hexa-	Total	%
4	0	0	0	0	78	55	133	9.17
5	0	0	360	62	4	0	426	29.38
6	0	214	265	14	0	0	493	34.00
7	0	49	66	0	0	0	115	7.93
8	0	27	4	0	0	0	31	2.14
9	0	23	2	0	0	0	25	1.72
10	0	18	0	0	0	0	18	1.24
11	0	18	0	0	0	0	18	1.24
12	65	1	0	0	0	0	66	4.55
≥12	125	0	0	0	0	0	125	8.62
Total	190	350	697	76	82	55	1450	100
%	13.10	24.14	48.07	5.24	5.66	3.79	100	

## Conclusion

Using Illumina sequencing technology, a large transcriptome dataset composed of 46,910 transcripts was achieved for *R. leptotubula*. Of these, 23,470 were annotated with gene descriptions from Nr, Swiss-Prot, COG and KEGG databases. Based on the assembled unigenes, 1,916 Pfam domains and 47 unigenes encoding Hsps were identified. A total of 1,450 SSRs were predicted. The platform constructed in this study is beneficial for us to have a better understanding of the fundamental molecular knowledge of this pest. It is also valuable for further research of gene expression, genomics, and functional genomics on this species.

## Supporting Information

Figure S1 **Amino acid alignment of predicted** ***Rhyacionia leptotubula*** **Hsp10 to that of other insect species.** Conserved residues are shaded. Abbreviations are the same as Figure 4.
(PPT)

Figure S2 **Amino acid alignment of predicted** ***Rhyacionia leptotubula*** **sHsp to that of other insect species.** Conserved residues are shaded. Abbreviations are the same as Figure 4.
(PPT)

Figure S3 **Amino acid alignment of predicted** ***Rhyacionia leptotubula*** **Hsp40 to that of other insect species.** Conserved residues are shaded. Abbreviations are the same as Figure 4.
(PPT)

Figure S4 **Amino acid alignment of predicted** ***Rhyacionia leptotubula*** **Hsp60 to that of other insect species.** Conserved residues are shaded. Abbreviations are the same as Figure 4.
(PPT)

Figure S5 **Amino acid alignment of predicted** ***Rhyacionia leptotubula*** **Hsp70 to that of other insect species.** Conserved residues are shaded. Abbreviations are the same as Figure 4.
(PPT)

Figure S6 **Amino acid alignment of predicted** ***Rhyacionia leptotubula*** **Hsp90 to that of other insect species.** Conserved residues are shaded. Abbreviations are the same as Figure 4.
(PPT)

Figure S7 **Amino acid alignment of predicted** ***Rhyacionia leptotubula*** **Hsp105/110 to that of other insect species.** Conserved residues are shaded. Abbreviations are the same as Figure 4.
(PPT)

Table S1 Top Blastx hits of the unigenes against NCBI Nr database with an E-value cut-off 10^−5^.
(XLS)

Table S2 The number of unigenes annotated in the public database.
(DOC)

Table S3 Pathway assignment based on KEGG.
(XLS)

Table S4 Pfam domain sereched in the unigenes.
(XLS)

Table S5 Detailed information of the designed primers.
(XLS)

Table S6 Randomly selected SSR markers used to validate the amplification.
(XLS)
