# Supplementary figures and images for: De novo Assembly and Characterization of the Global Transcriptome for Rhyacionia leptotubula Using Illumina Paired-End Sequencing
Source: PLoS One. 2013 Nov 21;8(11):e81096. doi: 10.1371/journal.pone.0081096 (PMC3837686; doi:10.1371/journal.pone.0081096)

## Slide 1
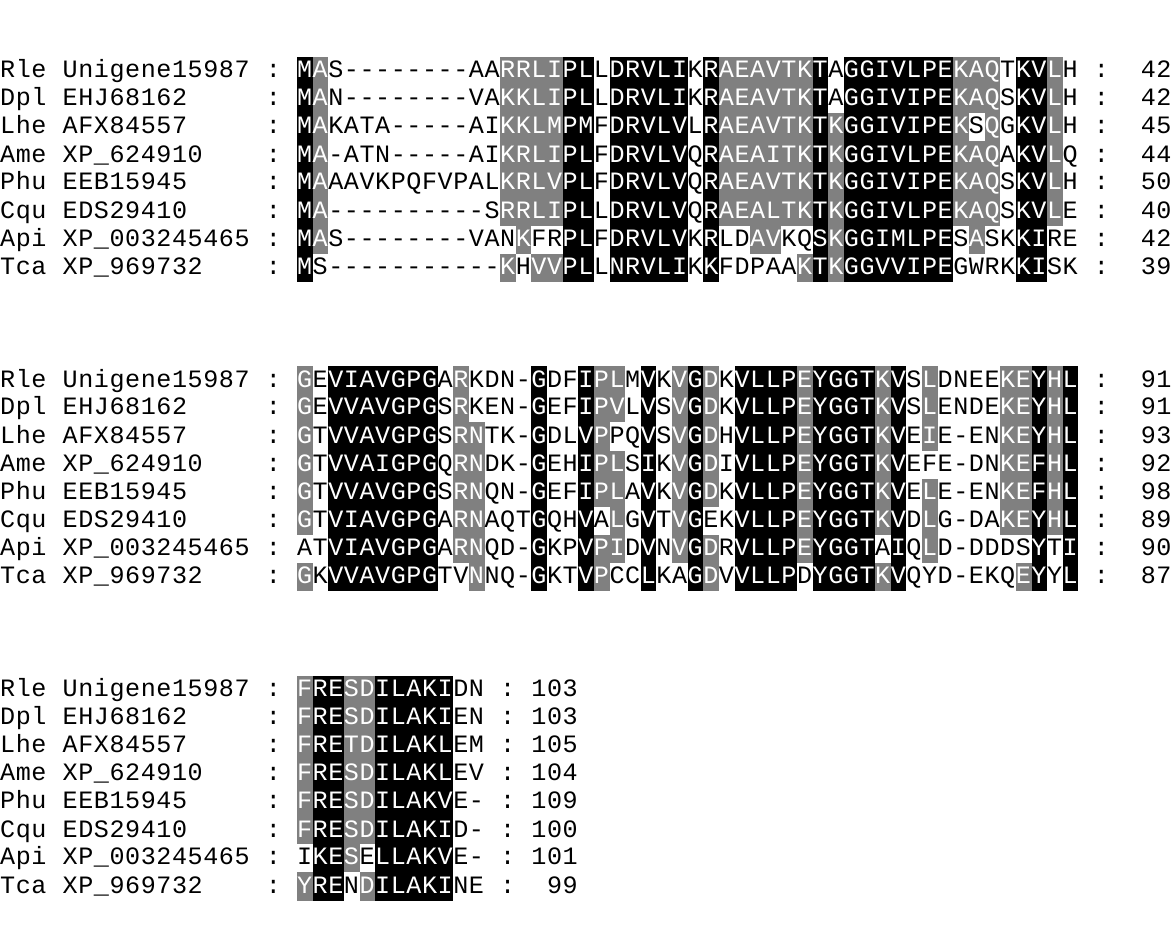

Supplement: Figure S1 — Amino acid alignment of predicted Rhyacionia leptotubula Hsp10 to that of other insect species. Conserved residues are shaded. Abbreviations are the same as Figure 4. (PPT) [file pone.0081096.s001.ppt]

## Slide 1
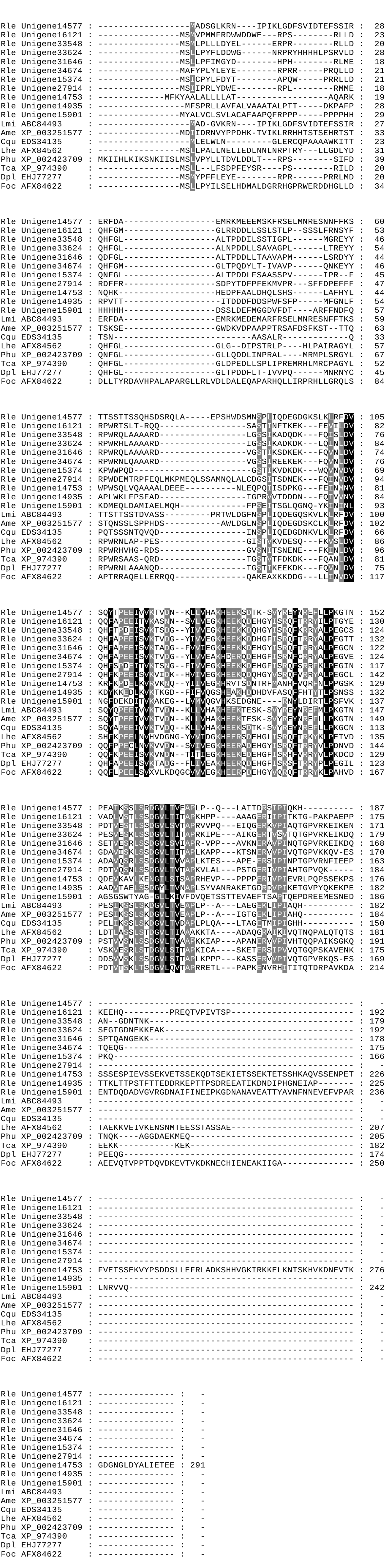

Supplement: Figure S2 — Amino acid alignment of predicted Rhyacionia leptotubula sHsp to that of other insect species. Conserved residues are shaded. Abbreviations are the same as Figure 4. (PPT) [file pone.0081096.s002.ppt]

## Slide 1
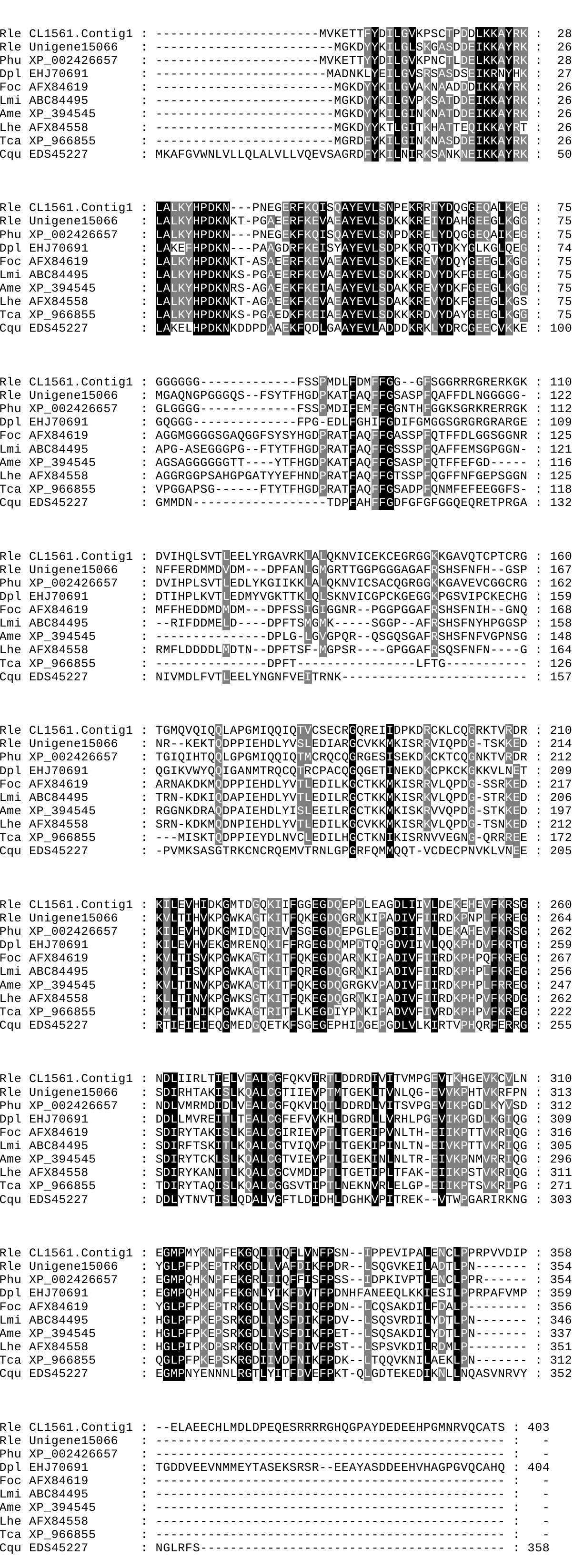

Supplement: Figure S3 — Amino acid alignment of predicted Rhyacionia leptotubula Hsp40 to that of other insect species. Conserved residues are shaded. Abbreviations are the same as Figure 4. (PPT) [file pone.0081096.s003.ppt]

## Slide 1
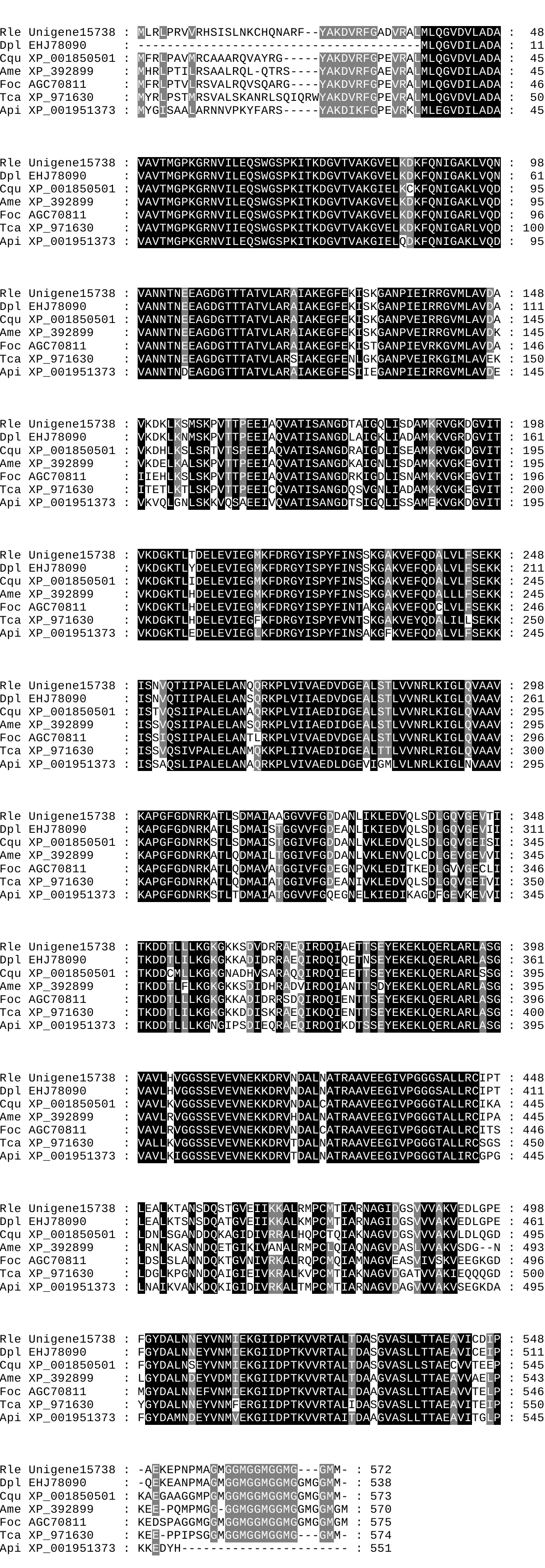

Supplement: Figure S4 — Amino acid alignment of predicted Rhyacionia leptotubula Hsp60 to that of other insect species. Conserved residues are shaded. Abbreviations are the same as Figure 4. (PPT) [file pone.0081096.s004.ppt]

## Slide 1
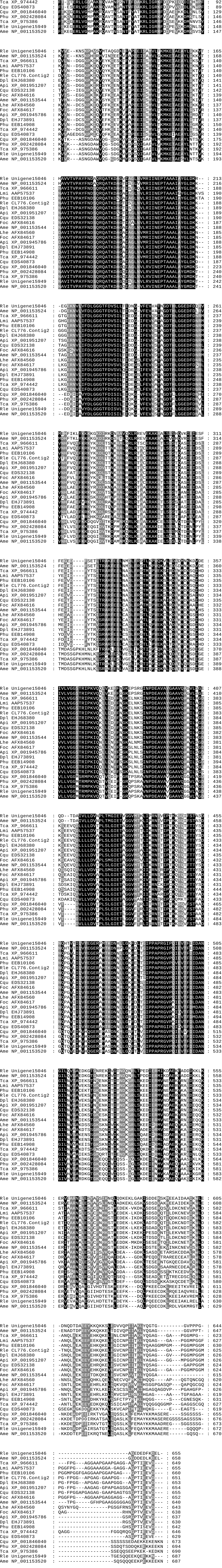

Supplement: Figure S5 — Amino acid alignment of predicted Rhyacionia leptotubula Hsp70 to that of other insect species. Conserved residues are shaded. Abbreviations are the same as Figure 4. (PPT) [file pone.0081096.s005.ppt]

## Slide 1
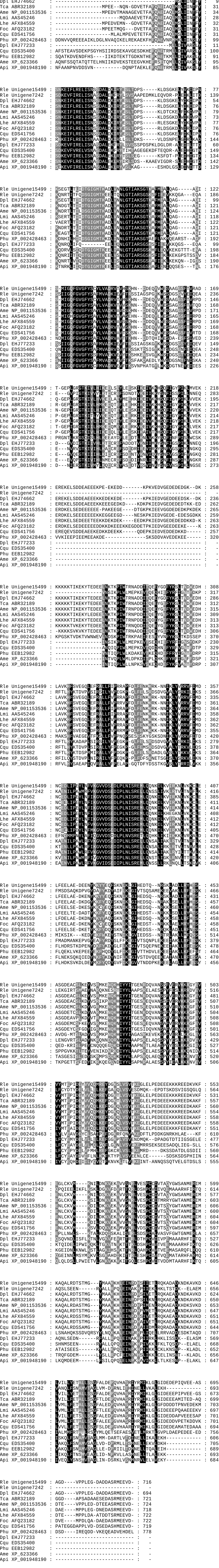

Supplement: Figure S6 — Amino acid alignment of predicted Rhyacionia leptotubula Hsp90 to that of other insect species. Conserved residues are shaded. Abbreviations are the same as Figure 4. (PPT) [file pone.0081096.s006.ppt]

## Slide 1
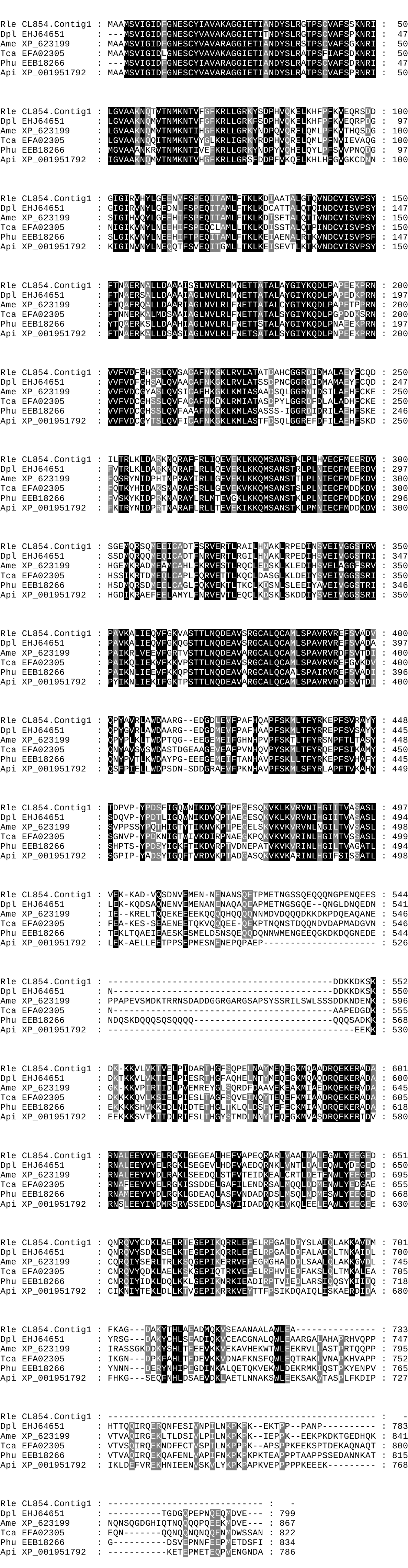

Supplement: Figure S7 — Amino acid alignment of predicted Rhyacionia leptotubula Hsp105/110 to that of other insect species. Conserved residues are shaded. Abbreviations are the same as Figure 4. (PPT) [file pone.0081096.s007.ppt]
