# Supplementary material for: De novo Assembly and Characterization of the Global Transcriptome for Rhyacionia leptotubula Using Illumina Paired-End Sequencing
Source: PLoS One. 2013 Nov 21;8(11):e81096. doi: 10.1371/journal.pone.0081096 (PMC3837686; doi:10.1371/journal.pone.0081096)
Supplement: Table S2 — The number of unigenes annotated in the public database. (DOC) [file pone.0081096.s009.doc]

**Table S2. The number of unigenes annotated in the public database.**

| **Annotation database** | **No of annotation** | **Percent of all unigenes** |
| --- | --- | --- |
| Nr | 22,581 | 48.14 |
| Nt | 7,778 | 16.58 |
| SwissProt | 15,621 | 33.30 |
| KEGG | 13,894 | 29.62 |
| COG | 6,937 | 14.79 |
| GO | 10,360 | 22.08 |
| All | 23,470 | 50.03 |
